# Quantifying annotation-driven bias in alternative splicing from EGAP metadata

**DOI:** 10.1093/nargab/lqaf141

**Published:** 2025-11-11

**Authors:** Rebeca de la Fuente, Wladimiro Díaz-Villanueva, Vicente Arnau, Andrés Moya

**Affiliations:** Foundation for the Promotion of Sanitary and Biomedical Research of the Valencian Community (FISABIO), 46020 Valencia, Spain; Foundation for the Promotion of Sanitary and Biomedical Research of the Valencian Community (FISABIO), 46020 Valencia, Spain; Institute of Integrative Systems Biology (I2Sysbio), University of Valencia and Spanish National Research Council (CSIC), 46980 Valencia, Spain; Center for Biomedical Research in Epidemiology and Public Health Network (CIBEResp), 28029 Madrid, Spain; Foundation for the Promotion of Sanitary and Biomedical Research of the Valencian Community (FISABIO), 46020 Valencia, Spain; Institute of Integrative Systems Biology (I2Sysbio), University of Valencia and Spanish National Research Council (CSIC), 46980 Valencia, Spain; Center for Biomedical Research in Epidemiology and Public Health Network (CIBEResp), 28029 Madrid, Spain; Foundation for the Promotion of Sanitary and Biomedical Research of the Valencian Community (FISABIO), 46020 Valencia, Spain; Institute of Integrative Systems Biology (I2Sysbio), University of Valencia and Spanish National Research Council (CSIC), 46980 Valencia, Spain; Center for Biomedical Research in Epidemiology and Public Health Network (CIBEResp), 28029 Madrid, Spain

## Abstract

Annotated coding sequences (CDSs) enable genome-wide estimates of alternative splicing. However, the quality and evidence support of these annotations can systematically bias estimates of splicing events across species. Here, we evaluate how annotation-related variables from the NCBI Eukaryotic Genome Annotation Pipeline affect inferred splicing levels. Analyzing 670 multicellular eukaryotes, we find that the percentage of CDSs supported by experimental evidence is the dominant predictor of variation in splicing estimates, whereas assembly quality and raw transcriptomic input play a minor role. To correct this annotation-driven bias, we introduce a normalization procedure based on polynomial regression, yielding an adjusted metric of alternative splicing. This novel metric preserves relative splicing complexity across species while mitigating annotation artifacts, with important implications for comparative genomics.

## Introduction

Alternative splicing is a key mechanism in eukaryotes that increases transcript and protein diversity by generating multiple mRNA (messenger RNA) isoforms from a single gene [1]. It plays fundamental roles in cell differentiation and speciation events [2–4], tissue-specific regulation [5], and developmental processes [6]. Also, by expanding the functional repertoire of the genome, alternative splicing allows for the fine-tuned regulation of complex processes like immune system signaling [7, 8]. In the nervous system, it has been shown to be essential for synapse formation and plasticity, providing molecular diversity required for the intricate functionality of the brain [9, 10]. Yet, our understanding of how alternative splicing contributes to transcriptomic and proteomic diversity across the eukaryotic tree of life is still limited. Barbosa-Morais *et al.* [11] found that within just 6 million years, the splicing profiles of physiologically equivalent organs have evolved to be more closely associated with the identity of a species than with the organ type. Notably, primate species exhibit a higher overall frequency of alternative splicing events compared to other vertebrates. Despite this rapid divergence, a small and evolutionarily conserved set of alternatively spliced exons maintains tissue-specific regulation across vertebrate lineages for over 350 million years, highlighting a core program of ancient splicing control that coexists with lineage-specific flexibility. These findings highlight the evolutionary plasticity of alternative splicing as a key mechanism shaping species-specific transcriptomic identities. However, only a limited number of studies have explored the evolutionary dynamics of alternative splicing beyond model organisms. Furthermore, most studies predominantly focus on specific tissues, or developmental stages, often under tightly controlled laboratory conditions. As a result, the current understanding of its regulation and function is largely context-specific and its generalizability across taxa remains limited [12].

Large-scale comparative analyses demand genome-wide measures that can capture transcript diversity across species. However, quantifying alternative splicing at the genome level remains a major challenge, and few metrics have been proposed to address this need. The most widely used measures focus on specific events, particularly through the PSI (Percent Spliced In) metric, which estimates the inclusion level of individual exons or splice junctions across samples [13, 14]. Other metrics include the number of transcript isoforms per gene [15], the fraction of multi-exonic genes that show splicing variation [11], and entropy-based diversity indices that capture the distribution of isoform usage across conditions or cell types [16, 17]. While these approaches provide precise measurements of isoform usage, they are often limited to species with high-quality RNA-seq datasets and lack a unified, genome-wide summary measure that enables cross-species comparisons. In response to these limitations, the scientific community has made increasing efforts to create unified annotation resources and cross-species frameworks. Initiatives such as GENCODE [18], Ensembl [19], and RefSeq [20] have progressively integrated multiple sources of experimental evidence to enhance transcriptome completeness and reduce annotation inconsistencies. In particular, the Eukaryotic Genome Annotation Pipeline (EGAP) from the NCBI integrates evidence from RNA-seq, expressed sequence tags (ESTs), and protein alignments, alongside computational predictions generated by tools such as Gnomon [21, 22]. The final annotation files generated by EGAP have a nonredundant set of genomic features, extensively annotated with structural and functional information, and enriched with cross-references to various annotation resources. These annotation efforts provide a basis for inferring transcript diversity at the species level from genome assemblies. In particular, we introduced the alternative splicing ratio (ASR), a genome-scale index derived from annotated coding sequences (CDSs), designed specifically to enable cross-species comparisons of splicing patterns [23].

While transcriptome assembly tools such as Trinity enable *de novo* isoform reconstruction in the absence of a reference genome [24], large-scale comparative analyses often rely on genome annotations to quantify splicing across diverse species. Although practical, genome-level metrics may also introduce potential biases, as annotation pipelines often rely on *ab initio* gene prediction, which infers gene structures directly from genomic sequence features such as splice sites, start and stop codons, and coding potential. While useful in the absence of transcriptomic data, *ab initio* predictions frequently fail to capture low-abundance isoforms, leading to underrepresentation of transcript diversity [25, 26]. Several recent studies have demonstrated that genome annotations can introduce systematic biases into transcriptome-based analyses. Notably, Zhang and Shao [27] showed that benchmarking the performance of transcriptome assemblers yields contradictory results depending on the choice of reference annotation—RefSeq versus Ensembl—despite using the same RNA-seq data. These discrepancies arise from differences in how transcripts are annotated, including variation in exon–intron boundaries, the inclusion or exclusion of retained introns, and differing thresholds for incorporating low-abundance or predicted isoforms. Such inconsistencies affect the apparent diversity and abundance of splice variants. Thus, this study provides evidence that annotation-derived splicing metrics can lead to divergent biological interpretations, even within well-characterized species. Consistent with this, Steijger *et al.* [28] showed that the accuracy of transcript reconstruction varies substantially depending on the reference annotation used. Furthermore, annotation databases tend to underrepresent lowly expressed isoforms, introducing a systematic bias in transcript-based metrics. Abascal *et al.* [29] demonstrated that alternatively spliced exons conserved across vertebrates are more likely to be annotated and translated, whereas weakly expressed isoforms often remain unannotated, even if biologically relevant. These findings underscore how annotation pipelines prioritize isoforms with strong empirical support, potentially skewing cross-species comparisons of splicing diversity. This annotation bias is further supported by Tardaguila *et al.* [26], who developed the SQANTI framework to classify transcript isoforms based on the type and quality of supporting evidence, revealing that annotations disproportionately favor long-read transcripts while systematically excluding partially supported variants. Tissue diversity and the level of experimental validation also tend to systematically underestimate isoform diversity, particularly in non-model species [25, 30]. These findings demonstrate that annotation-driven splicing metrics are influenced by multiple sources of bias, including not only the computational pipelines employed, but also the biological context and technical features of the underlying transcriptomic data. Despite these insights, few studies have systematically quantified how such biases impact global metrics of alternative splicing across species. In particular, it remains unclear to what extent species-level splicing estimates are shaped by features in the annotation files, such as the proportion of CDSs supported by experimental data, the depth and tissue diversity, or genome assembly quality. To address this gap, we analyzed the annotations of 670 multicellular eukaryotic genomes processed by the NCBI EGAP, showing that the proportion of CDSs fully supported by experimental evidence is the dominant predictor of splicing estimates.

## Materials and methods

### Data collection

We collected a dataset of annotated eukaryotic genomes from the NCBI, focusing specifically on assemblies curated by the RefSeq project [31]. In addition to the annotation files, each genome processed through EGAP is accompanied by a detailed report that provides extensive metadata on the source and type of evidence used for each predicted feature [32]. Annotation files and reports were retrieved in November 2024 from the NCBI FTP service, the official distribution platform for high-quality genomic resources [33]. Genome assemblies were filtered according to three criteria: (i) We restricted our dataset to assemblies annotated at the chromosome or complete genome level, which correspond to the highest-confidence genome builds. (ii) We restricted the dataset to assemblies from the RefSeq database that have been processed through the EGAP annotation pipeline, thereby ensuring consistency in annotation methodology across species. (iii) We focused exclusively on multicellular eukaryotes and grouped them into five major taxonomic categories. This resulted in a total of 694 species, classified into 133 mammals, 77 birds, 169 fish, 187 arthropods, and 128 plants. Taxonomic assignments were established based on a phylogenetic representation generated with the iTOL (Interactive Tree of Life) tool [34], and subsequently validated using the NCBI Taxonomy database [31, 35]. We parsed the RefSeq assembly summary file, applied the specified filters, and generated the species lists and metadata tables used for downstream analyses. All filtering, parsing, and data integration steps were implemented using custom Python scripts in Jupyter Notebook (v6.4.12) and are publicly available at https://github.com/sciencerdelafuente/AltSpliceLab.

### Annotation-related metrics

Tables 1–3 provide a conceptual description of the 23 metrics extracted from the NCBI annotation reports and considered in our evaluation of genome annotation quality. In Table 1, we summarize the set of metrics used to evaluate genome assembly quality [36]. Scaffold and contig counts, together with their corresponding N50 values, reflect the degree of assembly fragmentation. Higher N50 values and lower scaffold or contig counts indicate a more contiguous assembly, which is critical for accurate genome annotation. Gap length is also considered, as it serves as an indicator of assembly continuity, and has been previously recognized as a robust measure of assembly quality [37]. Table 2 summarizes the set of metrics corresponding to the molecular datasets that support the annotation process. This includes the number of protein and transcript sequences retrieved from the Entrez database, which are mapped to the genome assembly being annotated and used by Gnomon to gene prediction [20]. These sequences include mRNAs, ESTs, and curated protein records from RefSeq and other sources. Transcript sequences are aligned using Splign, while protein sequences are aligned using ProSplign, both tools developed by the NCBI [38]. The resulting alignments serve as evidence to support gene prediction. Gnomon, which is the *ab initio* gene prediction algorithm employed by EGAP, combines this external evidence with computational modeling to generate gene structures, including exon–intron boundaries and CDS annotations [22]. When high-confidence alignments occur, Gnomon uses them for predictions, prioritizing models that match the experimental data. In regions lacking strong evidence, Gnomon relies more heavily on intrinsic sequence signals to infer gene structure. Table 2 also includes metrics related to RNA-seq data attributes, such as the total number of reads, tissue diversity, and the number of experimental runs. Collectively, these variables serve as quantitative indicators of the extent of experimental support and annotation depth, allowing us to assess their influence on the genome annotation process [28, 39]. Finally, Table 3 summarizes the metrics related to the annotated CDSs, including those supported by experimental evidence and those inferred through computational prediction. Analyzing the impact of these metrics on alternative splicing levels will allow us to better understand the degree of reliance on predictive modeling and the balance between evidence-based and computational approaches [40, 41].

**Table 1.** Summary of annotation metadata metrics derived from the NCBI annotation reports

Metric	Description
**Number of gaps**	Total number of gaps present in the genome assembly
**Scaffold count**	Total number of scaffolds in the assembly
**Scaffold N50**	The length of the smallest scaffold such that 50% of the total genome length is covered by
	scaffolds of this length or longer
**Contig count**	Total number of contigs in the assembly
**Contig N50**	The length of the smallest contig such that 50% of the total genome length is covered by
	contigs of this length or longer
**Gap length**	Total length of gaps in the genome assembly
**Gap length (%)**	The percentage of the total genome length that consists of gaps

These metrics describe assembly-associated metadata, including scaffold and contig statistics, N50 values, and gap-related information. The metric for both scaffold N50 and contig N50, as well as the gap length, is measured in base pairs (bp).

**Table 2.** Summary of annotation metadata metrics associated with the molecular data supporting the annotation process

Metric	Description
**Number of proteins**	Total number of protein sequences retrieved from the Entrez database for the annotation process
**Number of transcripts**	Total number of transcript sequences retrieved from the Entrez database for the annotation process, including mRNA, ESTs, and other RNA sequences
**Number of proteins from** *Homo sapiens*	Total number of protein sequences originating from *H. sapiens* and retrieved from the Entrez database
**Number of transcripts from** *H. sapiens*	Total number of transcript sequences originating from *H. sapiens* and retrieved from the Entrez database
**Number of RNA-seq reads**	Total count of RNA-seq reads retrieved from the Sequence Read Archive (SRA) that were utilized during the annotation process to support gene prediction
**Number of tissues**	The number of distinct tissue types represented in the RNA-seq data
**Number of runs**	Total count of individual RNA-seq experimental datasets (runs), each corresponding to a specific biological sample, condition, or replicate

The table includes the number of proteins and transcripts retrieved from the Entrez database, and RNA-seq data characteristics, including read counts, tissue diversity, and experimental runs.

**Table 3.** Summary of annotation metadata metrics describing the number of CDSs generated during the annotation process, including total counts, those derived from computational models, and those supported by experimental evidence

Metric	Description
**CDSs**	Total number of CDSs annotated in the genome
**CDSs from model**	Total number of CDSs predicted by computational models, such as *ab initio* methods or guided by evidence but not experimentally validated
**Fully supported CDSs**	Total number of CDSs supported by direct experimental evidence, such as RNA-seq or protein alignments
**Known CDSs**	Number of CDSs that correspond to sequences already present in curated databases like RefSeq. These are derived from previously validated annotations
**CDSs with ${>}$ 5%** *ab initio*	Total number of CDSs where >5% of their bases are annotated using *ab initio* methods. This indicates regions where computational predictions were necessary due to insufficient experimental evidence
**CDSs from model (%)**	The percentage of total CDSs that are derived from computational models
**Known CDS (%)**	The percentage of total CDSs that are known from curated databases
**Fully supported CDSs (%)**	The percentage of total CDSs supported by experimental evidence
**CDSs with $>$ 5%** *ab initio * **(%)**	The percentage of total CDSs where >5% of their bases rely on *ab initio* predictions

### Alternative splicing ratio

**Figure 1.**
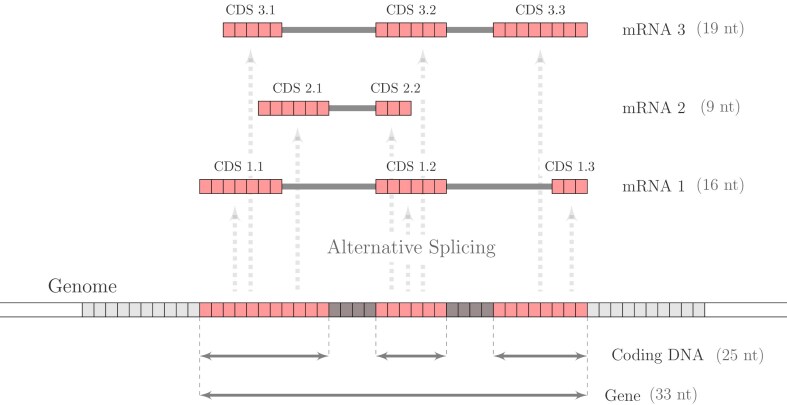
A toy gene model consisting of 33 nucleotides undergoes alternative splicing, generating three distinct mRNA isoforms of lengths 16 (mRNA1), 9 (mRNA2), and 19 (mRNA3) nucleotides, respectively. The combined coding DNA, corresponding to the genomic regions transcribed into these isoforms, spans 25 nucleotides. The ASR is calculated as the cumulative sum of isoform nucleotide lengths divided by the length of the coding DNA, yielding $\mathrm{ ASR}$ = (16 + 9 + 19) / 25 = 1.76. This figure was originally presented in [23].

A novel genome-scale metric of alternative splicing was proposed in [23] for the comparative study of splicing patterns across the tree of life. We refer to this metric as the *alternative splicing ratio*, which measures the average number of distinct transcripts generated per CDS. As illustrated in Fig. 1, which was previously presented in [23], we summarize the alternative splicing into a single numerical value. The figure provides a simplified example that schematically represents how alternative isoforms are generated from a gene. Although the metric can be computed for a single gene, the ASR can be generalized at the genome level by aggregating all annotated CDSs from transcripts and mapping them to their corresponding genomic loci. This yields a single summary index that reflects the extent of CDS reuse across the transcriptome or proteome, enabling standardized comparisons of alternative splicing complexity among species. Thus, higher values of ASR reflect a greater reuse of coding DNA across multiple transcript isoforms, whereas low ASR values reflects a near one-to-one mapping between transcripts and coding regions.

The ASR is computed from the RefSeq GFF annotation files. For each species, we parsed the corresponding annotation file and extracted information about all protein-coding genes and their CDSs. The files are organized using a parent–child structure, where genes act as parent elements to their corresponding transcripts (mRNAs) and each transcript is further linked to its CDSs. This hierarchical format reflects the biological relationship between a gene and its alternative isoforms, with each mRNA representing a distinct transcript variant. The CDSs associated with each mRNA define the protein-coding regions retained after splicing, ultimately specifying a unique protein isoform. In our analysis, we focused exclusively on CDSs with clearly defined hierarchical links, excluding pseudogenes and duplicated genes. The annotation files are structured in tab-separated tables, where each row corresponds to a genomic feature—such as a gene, mRNA, or CDS—and each column contains specific attributes, including unique identifiers, genomic positions, and functional annotations. For each genomic feature, we extracted its start and end coordinates, which allowed us to calculate the length of individual regions, such as genes, transcripts, and CDSs. Thus, the total gene content was quantified as the number of base pairs that fall within gene intervals. Similarly, coding size was computed by summing the nucleotides in the genome annotated as CDS.

Although the ASR was computed for all 694 species using their corresponding genome annotation files, annotation reports were only available for a subset of 670 species. As a consequence, the analysis of variables potentially influencing alternative splicing estimates was restricted to those species for which complete annotation metadata was successfully retrieved.

### Statistical analyses

We performed Spearman correlation analyses using R (version 4.3.3; [42]) to quantify pairwise associations between the ASR and annotation-related variables. Correlation coefficients were computed using the *cor()* function with method set to “spearman,” which captures monotonic relationships and is robust to nonlinear trends. Correlation matrices were generated across the different variables, serving as the basis for subsequent multivariate analyses and variable selection.

We then conducted a multivariate analysis to evaluate the joint contribution of annotation variables to ASR variation. To reduce multicollinearity and prioritize informative predictors, we applied LASSO (Least Absolute Shrinkage and Selection Operator) regression—a regularized linear modeling technique well suited for high-dimensional data [43]. The dataset was randomly partitioned into a training set (80%) and a test set (20%) using the *createDataPartition()* function from the *caret* package [44]. The LASSO model was fitted to the training data using the *glmnet* package in R [45], and the optimal penalty parameter $\lambda$ was selected via 10-fold cross-validation using *cv.glmnet()*, minimizing the mean squared error. The final model was then applied to the test set to evaluate predictive performance. To interpret the results, we extracted the nonzero coefficients from the best-fitting model and visualized their magnitude as an indication of variable importance.

### Empirical validation using long-read data

We performed an empirical validation of the ASR metric using long-read RNA sequencing data from *Mus musculus*. A subset of publicly available transcriptomic datasets was retrieved from the NCBI SRA, applying the following filters: (”Mus musculus”[Organism]) AND (”RNA-Seq”[Strategy] OR ”Iso-Seq”[Strategy] OR ”FL-cDNA”[Strategy]) AND (”PacBio SMRT”[Platform]), further restricted to single-run experiments [46]. These datasets reflect high-quality transcript models and capture full-length isoforms. The *M. musculus* reference genome was obtained from RefSeq. Genome indexing was performed using Minimap2 (v2.24; [47]). For each sample, transcript alignment was also performed using Minimap2, aligning FASTQ files to the reference genome. GTF-like annotations were generated using StringTie2 (v2.2.1), which reconstructs transcript structures directly from aligned long-read data [48]. Each resulting GTF file contains the expressed isoforms per sample and served as input for ASR calculation. Each resulting GTF file contains the expressed isoforms per sample and served as input for ASR calculation. The scripts developed to perform these analyses—including transcriptome alignment, GTF generation, and ASR computation—are publicly available in the associated GitHub repository: https://github.com/sciencerdelafuente/AltSpliceLab.

## Results

### Associations between annotation metadata and alternative splicing

In this section, we performed correlation analyses to examine the influence of 23 annotation-related quality variables on ASR estimates. In the first block of Table 4 and Supplementary Fig. S1, we observe weak correlations between the ASR and assembly-related metrics, with the strongest correlation found with contig N50 ($\rho =0.322$, *P*-value $<.001$). These results suggest that more fragmented assemblies may slightly reduce the number of alternative isoforms detected, but the weakness of the correlations indicates that it may not play a major role in shaping alternative splicing estimates. Despite potential confounding factors, the absence of strong associations supports the conclusion that assembly quality has limited impact on ASR quantification. Pairwise correlations among assembly metrics were also evaluated. The number of gaps shows a strong positive correlation with gap length ($\rho =0.758$, *P*-value $<.001$) and a strong negative correlation with contig N50 ($\rho =-0.731$, *P*-value $<.001$). In turn, total gap length shows a strong association with the proportion of gaps relative to genome size ($\rho =0.965$, *P*-value $<.001$). Scaffold and contig counts were positively correlated ($\rho =0.980$, *P*-value $<.001$), supporting the interpretation that assemblies with many scaffolds also have numerous small contigs, indicating low contiguity. These results confirm that metrics such as gap count, total gap length, and scaffold/contig N50 provide reliable indicators of assembly continuity, although they show limited correlation with ASR levels.

**Table 4.** Pairwise Spearman correlation matrices between the ASR and the annotation-related metrics described in Tables 1–3

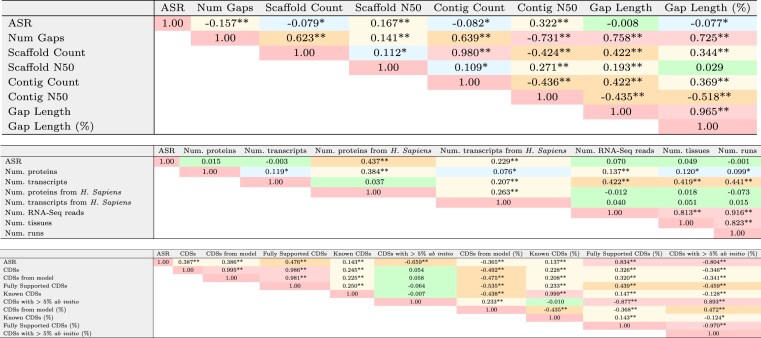

The first subtable displays correlations between ASR and assembly quality metrics (Table 1); the second includes correlations with experimental support metrics (Table 2); and the third corresponds to CDS-related annotation output metrics (Table 3). Asterisks indicate significance levels: $P < .05$ (*), $P < .001$ (**). Cell colors reflect the strength of the correlation coefficients.

We evaluated pairwise Spearman correlations between ASR and experimental evidence variables (Table 2), as summarized in the intermediate block of Table 4 and Supplementary Fig. S2. No significant correlation was observed between ASR and the total number of proteins ($\rho = 0.015$, *P*-value $>.05$) or transcripts ($\rho = -0.003$, *P*-value $>.05$) from the Entrez database. A weak but statistically significant correlation was detected between ASR and the number of proteins ($\rho = 0.437$, *P*-value $<.001$) and transcripts ($\rho = 0.229$, *P*-value $<.001$) derived from *H. sapiens*. Notably, no significant correlations were observed between ASR and the number of RNA-seq reads, tissue diversity, or the number of sequencing runs. These findings indicate that ASR estimates are largely unaffected by overall experimental evidence volume, with the exception of a minor influence from human-derived data. Correlations among evidence-derived variables were also assessed to explore interdependencies. Strong correlations were observed among RNA-seq read count, tissue diversity, and number of sequencing runs ($\rho > 0.8$, *P*-value $<.001$), reflecting typical features of RNA-seq experimental design.

In contrast to experimental evidence variables, which showed little correlation with ASR values, all annotation-derived CDS variables demonstrated consistent associations. This highlights the significant role of the annotation process in determining observed alternative splicing complexity. As shown in the final block of Table 4 and in Supplementary Fig. S3, correlations between ASR and CDS-related annotation metrics range from very weak to strong. Specifically, very weak correlations were found for the number ($\rho = 0.143$, *P*-value $<.001$) and proportion of known CDSs ($\rho = 0.137$, *P*-value $<.001$). Weak correlations were detected with the total number of CDSs ($\rho = 0.387$, *P*-value $<.001$), the number of model-derived CDSs ($\rho = 0.386$, *P*-value $<.001$), and their proportion ($\rho = -0.365$, *P*-value $<.001$). Moderate correlations were observed for the number of fully supported CDSs ($\rho = 0.476$, *P*-value $<.001$) and for CDSs with >5% *ab initio* content ($\rho = -0.659$, *P*-value $<.001$). Strong correlations were found with both the percentage of fully supported CDSs ($\rho = 0.834$, *P*-value $<.001$) and the proportion of CDSs with high *ab initio* contribution ($\rho = -0.804$, *P*-value $<.001$). These results suggest that splicing estimates are more influenced by the effectiveness of evidence integration during annotation than by the absolute volume of input data. In particular, the percentage of fully supported CDSs serves as a proxy for the degree to which empirical data are reflected in isoform diversity.

Several CDS-related annotation metrics were strongly correlated, indicating a high degree of redundancy among these features. Specifically, the number of known CDSs was highly correlated with its corresponding proportion ($\rho = 0.999$, *P*-value $<.001$), while the total CDS count showed strong associations with both fully supported ($\rho = 0.986$, *P*-value $<.001$) and model-derived CDSs ($\rho = 0.995$, *P*-value $<.001$). This interdependence reflects the design of the annotation pipeline (e.g. Gnomon), which combines *ab initio* predictions with transcript and protein alignments. As such, fully supported and model-derived CDSs are not mutually exclusive but represent overlapping strategies, often applied to the same transcript. Strong correlations were also observed among fully supported CDSs (%), CDSs with >5% *ab initio*, and their corresponding proportion ($|\rho | > 0.8$, *P*-value $<.001$), yet these do not correlate with the absolute number of fully supported CDSs ($|\rho | < 0.5$, *P*-value $<.001$).

**Figure 2.**
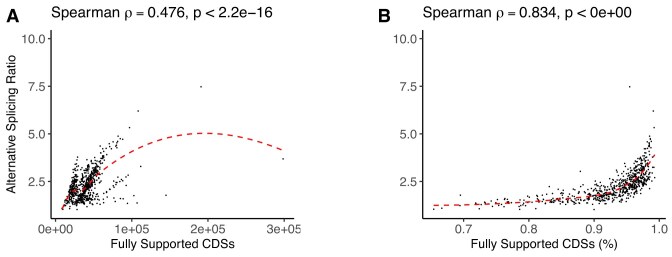
Spearman correlation between ASR and annotation metrics related to CDSs. (**A**) Correlation between ASR and the absolute number of fully supported CDSs. (**B**) Correlation between ASR and the percentage of fully supported CDSs. These variables, which are among the most relevant predictors of splicing estimates, are described in detail in Table 3.

As illustrated in Fig. 2, the correlation between the ASR and the absolute number of fully supported CDSs exhibits considerable dispersion and suggests the presence of multiple regimes across species. In contrast, when alternative splicing values are plotted against the percentage of fully supported CDSs, the relationship becomes much more consistent and exhibits a smooth, nonlinear trend with minimal scatter. This contrast indicates that the relative proportion of empirical evidence within the annotation—rather than its absolute amount—better explains the observed levels of alternative splicing. Accordingly, the percentage of fully supported CDSs serves as a normalization factor, capturing the extent to which empirical evidence contributes to isoform diversity. These findings reinforce the view that annotation-based metrics like ASR are intrinsically shaped by the architecture of the annotation process, emphasizing the importance of normalization strategies to mitigate annotation-driven biases.

A multivariate analysis was conducted to identify the most influential predictors of alternative splicing variability (see the “Materials and methods” section). A LASSO (Least Absolute Shrinkage and Selection Operator) regression was applied as a regularization technique to select a minimal subset of predictive variables while penalizing model complexity. This approach allowed the detection of interdependencies among predictors that may remain obscured in univariate analyses. By considering all variables simultaneously, the analysis revealed which factors most strongly contribute to variation in splicing levels. A subset of eight representative variables was selected based on the interdependencies identified in previous analyses. This selection aimed to retain the relevant predictors while minimizing collinearity and redundancy. The variables included were contig N50, scaffold N50, number of proteins from *H. sapiens*, number of transcripts from *H. sapiens*, number of RNA-seq reads, fully supported CDSs (%), model-derived CDSs (%), and known CDSs (%).

**Figure 3.**
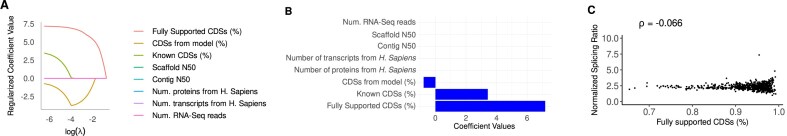
LASSO regression results for predicting ASR. (**A**) Evolution of model coefficients as the regularization parameter ($\lambda$ ) increases, illustrating how less informative variables are progressively excluded from the model. Only three predictors remain with nonzero coefficients at high penalization levels: fully supported CDSs (%), known CDSs (%), and CDSs from Model (%). (**B**) Final coefficient values using the optimal penalty of $\lambda$, which achieves a balance between model simplicity and predictive power. (**C**) Spearman correlation between the normalized splicing ratio (ASR$^{*}$) and the percentage of fully supported CDSs, which shows a nonsignificant association after normalization.

Figure 3A displays the evolution of the LASSO model as the regularization parameter ($\lambda$) increases. At lower values of $\lambda$, the model retains a larger set of predictors, including some that contribute marginally to the explained variance in splicing. As $\lambda$ increases, the model imposes stronger penalties on less informative variables, progressively shrinking their coefficients toward zero. This results in a more compact model that retains only the most relevant predictors. In this analysis, only three variables remained as key predictors: fully supported CDSs (%), known CDSs (%), and CDSs derived from models (%). These variables remain relevant for predicting alternative splicing even under strong penalization, while the remaining predictors are excluded from the model altogether. As a consequence, although many variables are correlated with splicing levels, only a few provide independent, nonredundant information.

In the pairwise correlation analyses, the percentage of CDSs derived from the model and the percentage of known CDSs showed only weak correlations with ASR. Interestingly, despite their weak pairwise associations, these two variables emerged as among the most predictive in the multivariate LASSO model. This indicates that they may capture complementary aspects of the annotation process that become informative when considered in combination with other variables. In contrast, the percentage of fully supported CDSs showed a very strong correlation with ASR values. Consistently, this variable also emerged as the most predictive in the multivariate model, indicating that the evidence support of annotated CDSs may systematically bias ASR estimates. Species with higher proportions of fully supported CDSs tend to show elevated levels of alternative splicing, potentially reflecting differences in annotation quality rather than true biological variation.

Using the optimal penalization parameter ($\lambda$) selected during LASSO model fitting, the contribution of each annotation feature to ASR prediction was quantified (Fig. 3B). The final model retained three annotation variables—percentage of fully supported CDSs, known CDSs, and model-derived CDSs—all exhibiting nonzero coefficients. Among these, the percentage of fully supported CDSs had the largest coefficient, indicating it was the most influential predictor of ASR. All other variables had coefficients near zero, indicating a negligible impact on the model. Finally, we evaluated how well the model could predict ASR values in a separate dataset that was not used during model training. The results showed a good level of predictive accuracy, with the model explaining ~60% of the variation in ASR values. These findings support the conclusion that the selected annotation-derived variables capture a substantial proportion of the interspecies variability in splicing complexity.

Since the percentage of fully supported CDSs was identified as the main driver of annotation-related biases in ASR, we normalized ASR values based on its relationship with this variable. This approach allowed us to account for variation that may stem from differences in annotation quality. To capture the nonlinear pattern of this association, which is illustrated in Fig. 2B, we fitted a fourth-degree polynomial regression model. The model provided a good fit to the data, explaining ~60% of the variability in ASR values (multiple and adjusted $R^2 = 0.602$). The estimated coefficients for all polynomial terms (up to the fourth degree), along with their statistical significance, are presented in Table 5. Thus, we normalized ASR values according to the following formulation:


(1) \begin{eqnarray*}
\mathrm{ ASR}^{\text{*}} = \mathrm{ ASR}_{\mathrm{ obs}} - \mathrm{ ASR}_{\mathrm{ exp}} + \sigma _{\mathrm{ min}},
\end{eqnarray*}

where $\mathrm{ ASR}_{\mathrm{ obs}}$ represents the observed alternative splicing values computed from the annotated files and $\mathrm{ ASR}_{\mathrm{ exp}}$ corresponds to the expected values derived from the fitted polynomial model:


(2) \begin{eqnarray*}
\mathrm{ ASR}_{\mathrm{ exp}} = \beta _0 + \beta _1 x + \beta _2 x^{2} + \beta _3 x^{3} + \beta _4 x^{4},
\end{eqnarray*}

where $x$ represents the percentage of fully supported CDSs and the $\beta _i$ are the estimated coefficients reported in Table 5. The difference between the observed and expected values centers the data around zero, highlighting deviations as positive (above expected) or negative (below expected). However, values below 1 ($\mathrm{ ASR}^{\text{*}}<1$) lack biological meaning. Thus, we added a normalization constant


(3) \begin{eqnarray*}
\sigma _{\mathrm{ min}}= 1 - \mathrm{ min} {\lbrace \mathrm{ ASR}^{i}_{\mathrm{ obs}} - \mathrm{ ASR}^{i}_{\mathrm{ exp}}\rbrace }_{i},
\end{eqnarray*}

which adjusted all values so that the minimum normalized $\mathrm{ ASR}^{\text{*}}$ is equal to 1. This correction resulted in a normalized measure, ASR$^{*}$, which preserves relative differences in alternative splicing while reducing biases introduced by annotation evidence. As a result, this metric allows for more accurate cross-species comparisons and provides a more reliable basis for downstream analyses of splicing complexity. As shown in Fig. 3C, and as expected after normalization, the correlation between ASR$^{*}$ and the percentage of fully supported CDSs becomes nonsignificant, with a coefficient close to zero. Accordingly, the normalization substantially eliminates the correlation between ASR and annotation support (see Fig. 3C), effectively correcting for the EGAP-related bias. However, it is important to emphasize that these normalized values should be interpreted as representative estimates rather than precise measurements, given that the normalization process is based on an empirical approach.

**Table 5.** Coefficients of the fourth-degree polynomial regression model used to describe the relationship between ASR and the percentage of fully supported CDSs

Term	Estimate ($\beta$)	Standard error	*t*-value	*P*-value
Intercept	2.3180	0.0185	125.28	$< 2 {\times} 10^{-16}$
Degree 1 (linear term)	12.8359	0.4789	26.80	$< 2 {\times} 10^{-16}$
Degree 2 (quadratic term)	6.9187	0.4789	14.45	$< 2 {\times} 10^{-16}$
Degree 3 (cubic term)	4.0667	0.4789	8.49	$< 2 {\times} 10^{-16}$
Degree 4 (quartic term)	2.1580	0.4789	4.51	$7.81 {\times} 10^{-6}$

The table includes the estimated value, standard error, t-value, and P-value for each polynomial term. All terms are statistically significant, indicating that higher-order components contribute meaningfully to capturing the nonlinear association.

### Validation

The pipeline prioritizes curated RefSeq transcripts and genomic sequences when available, using them to directly annotate CDSs or to guide predictions. However, most annotations are based on short-read RNA-seq data, which cannot provide information about the exact transcript being expressed in a sample. Addressing this requires long-read sequencing, which is used in the EGAP but not extensively. Additionally, mappings from long reads are not reported in the annotation files, and while individual features include counts of supporting samples, the reports do not provide a detailed list of the samples associated with each feature.

To empirically validate the ASR metrics, we analyzed a curated subset of long-read RNA sequencing datasets from *M. musculus*, selected from the NCBI SRA. For each sample, transcriptomes were aligned to the *M. musculus* reference genome, and splicing metrics were derived directly from the resulting annotations. These empirically derived ASR values were then compared to the annotation-derived ASR and ASR* estimates previously obtained from the RefSeq database, which had been annotated using the NCBI EGAP pipeline.

**Figure 4.**
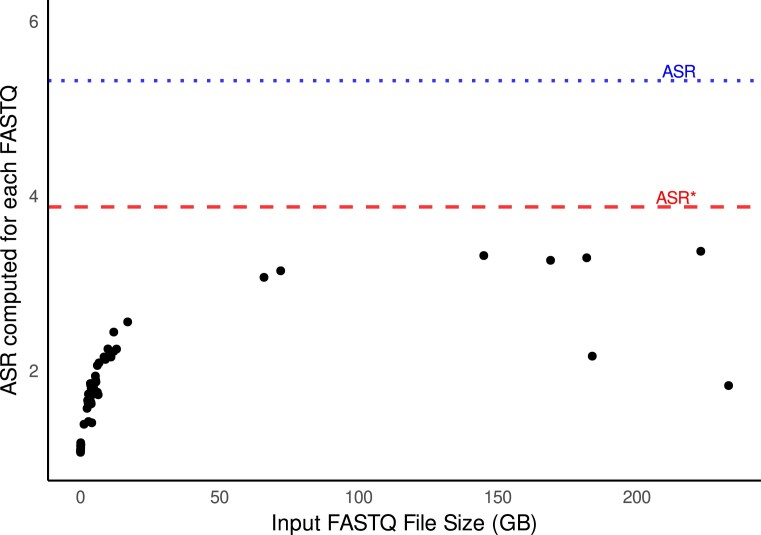
Relationship between input FASTQ file size and ASR across individual sequencing runs. Each point represents the ASR computed for a single FASTQ input. Two reference thresholds are shown: the blue dotted line represents the ASR threshold (5.327) and the red dashed line indicates the normalized ASR* baseline (3.882), both derived from previously computed values using the NCBI’s EGAP annotation pipeline applied to RefSeq genome assemblies.

As observed in Fig. 4, the comparative analysis revealed a consistent pattern between empirical and annotation-derived ASR estimates, supporting the biological relevance of our proposed metric. Notably, the normalized ASR* values demonstrated consistency in reflecting splicing complexity, aligning closely with patterns observed in empirical long-read datasets. This validation step confirms that ASR and ASR* are not only computational descriptors extracted from annotation files but also approximate well the splicing richness observed in empirical long-read transcriptomic data.

## Discussion

Comparative analyses of alternative splicing across species are challenged by variability in genome annotation quality. This issue has been broadly acknowledged in large-scale studies [11, 49], but the field still lacks standardized metrics to quantify and correct annotation-driven biases. Our study addresses this gap by systematically analyzing 23 genome assemblies and annotation variables across 670 eukaryotic species and introducing a normalized splicing metric that adjusts for disparities in annotation support.

While it is well known that empirical transcriptomic evidence improves gene annotation accuracy [40], we show that one specific variable—the percentage of fully supported CDSs—acts as a dominant driver of variation in splicing estimates across genomes. Although this finding may not be unexpected, our analysis is the first to systematically quantify the effect across hundreds of eukaryotic species, and correct for it using a normalized ASR metric (ASR*). Previous efforts such as GENCODE [50] or the RefSeq EGAP pipeline [20] have noted differences between evidence-supported and computationally predicted transcripts, but did not directly assess their impact on downstream transcriptomic metrics.

To correct the annotation bias, we fitted a fourth-degree polynomial regression model and developed a normalized ASR metric, which subtracts the expected splicing value conditioned on empirical support and recenters values. This normalization eliminated the correlation between ASR and annotation quality, enabling more accurate cross-species comparisons of splicing complexity. Although normalization techniques have long been used in transcriptomics [51–53], applying a support-based correction at the annotation level provides a novel extension for comparative genomics.

Finally, to validate our model empirically, we analyzed long-read RNA sequencing datasets from *M. musculus*, which offer complete transcript models and improved isoform resolution compared to short-read methods [54]. The empirical ASR values derived from these datasets were broadly consistent with the normalized value, ASR*. This supports the interpretation that ASR* is not only a computational artifact but reflects underlying splicing diversity captured by empirical data. In sum, although the existence of annotation bias is not novel, our work presents a reproducible framework to quantify and correct its effects. Our findings reinforce the importance of interpreting splicing estimates through the lens of annotation evidence, and suggest that metrics such as ASR* can help decouple technical artifacts from true biological variation.

Beyond demonstrating the existence of annotation-driven bias, our study provides a practical framework that can be directly integrated into genome annotation pipelines. Resources such as RefSeq could use the ASR* metric to routinely monitor how annotation evidence impacts transcript diversity estimates. This would allow to identify assemblies where low empirical support is likely to distort isoform representation. More broadly, quantifying and correcting for annotation bias is essential for researchers comparing transcriptomes across taxa. We anticipate that integrating this approach into annotation workflows would improve the robustness of comparative genomics studies.

## Supplementary Material

lqaf141_Supplemental_File

## Data Availability

The full list of analyzed species, along with all scripts, metadata tables, and processed annotation files used in this study are available at https://github.com/sciencerdelafuente/AltSpliceLab..
