# Supplementary material for: Quantifying annotation-driven bias in alternative splicing from EGAP metadata
Source: NAR Genom Bioinform. 2025 Nov 11;7(4):lqaf141. doi: 10.1093/nargab/lqaf141 (PMC12605758; doi:10.1093/nargab/lqaf141)
Supplement: lqaf141_Supplemental_File [file lqaf141_supplemental_file.pdf]

Supplementary Material

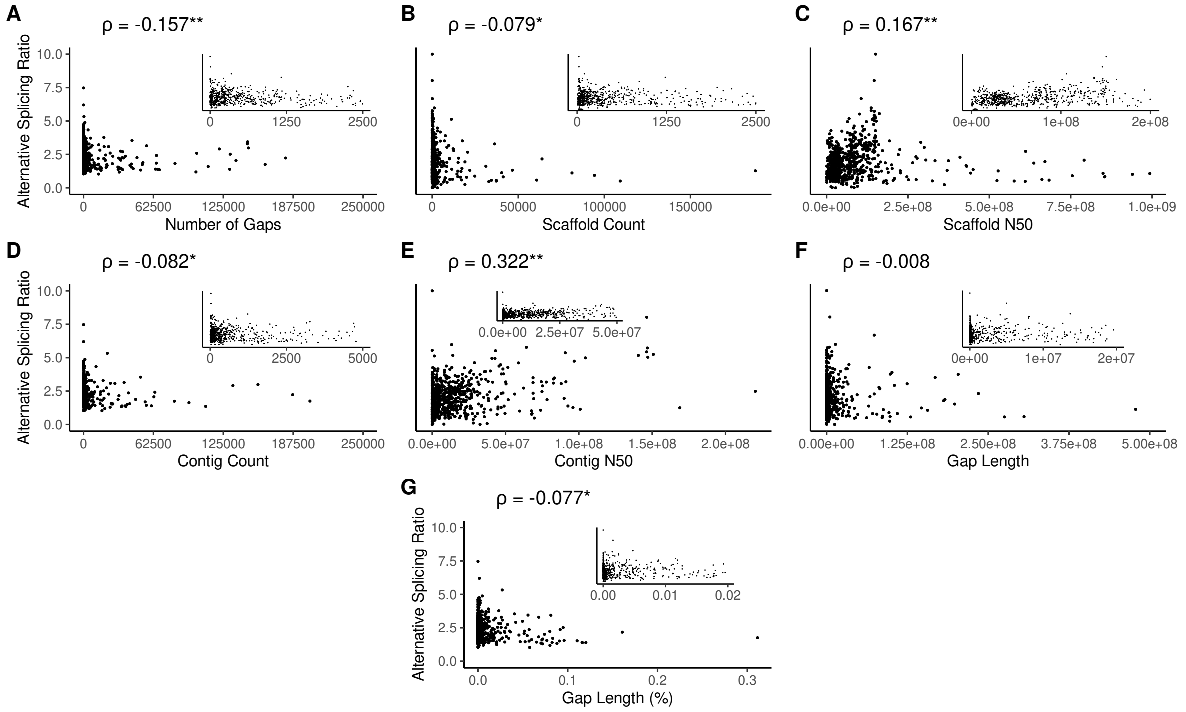

Supplementary Figure 1. Spearman correlation between the alternative splicing ratio and the genome assembly quality metrics: number of gaps, scaffold count, scaffold N50, contig count, contig N50, gap length, and gap length (%).

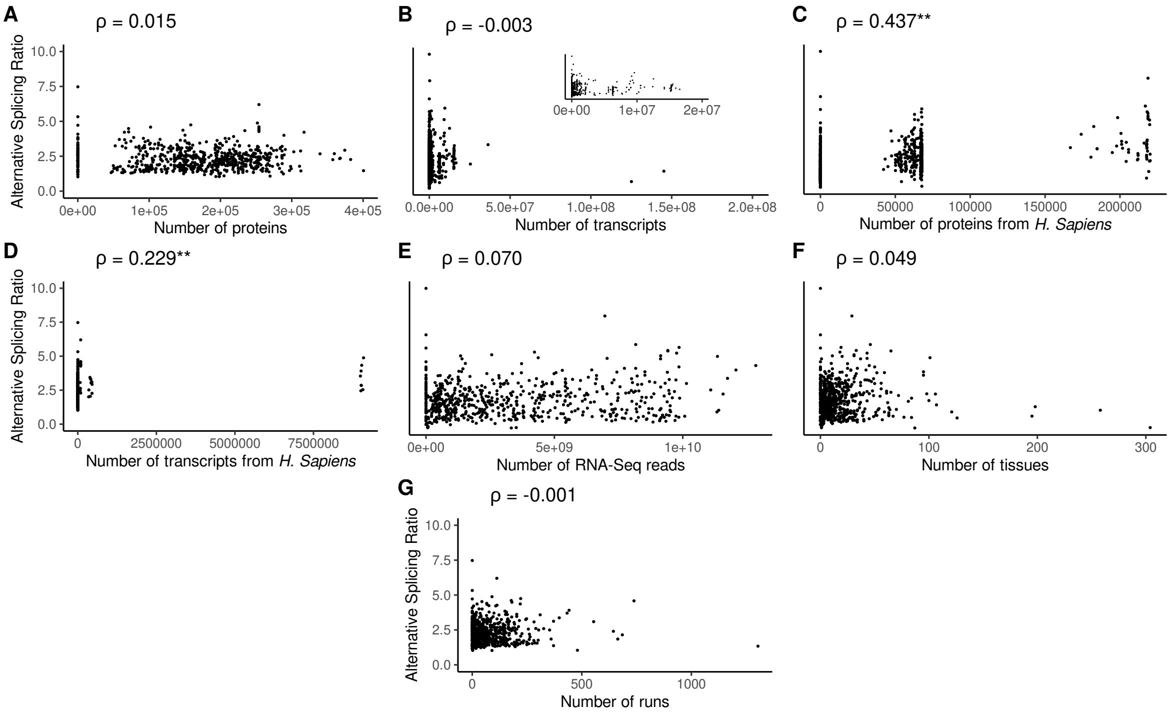

Supplementary Figure 2. Spearman correlation between the alternative splicing ratio and the experimental evidence metrics: number of proteins, number of transcripts, number of proteins from *Homo sapiens*, number of transcripts from *Homo sapiens*, number of RNA-Seq reads, number of tissues, and number of experimental runs.

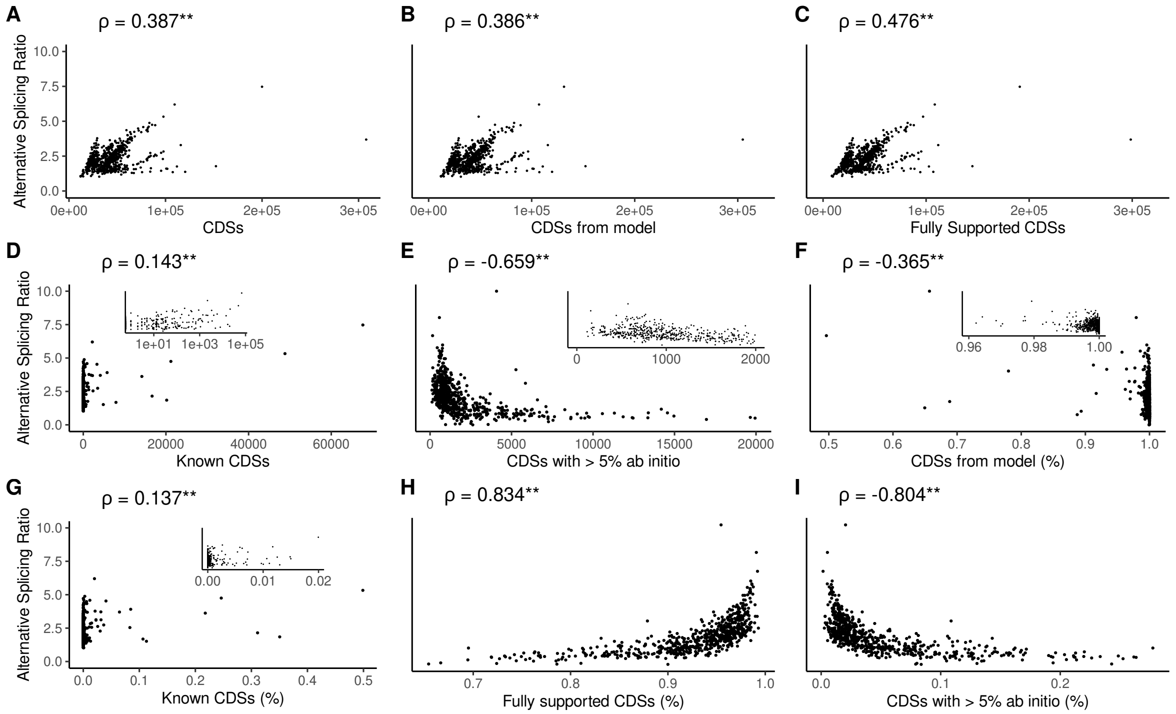

Supplementary Figure 3. Spearman correlation between the alternative splicing ratio and the CDS-related annotation metrics: total number of CDSs, number of CDSs from model, number of fully supported CDSs, number of known CDSs, number of CDSs for which more than 5% of their sequence is annotated using ab initio prediction, percentage of CDSs from model, percentage of known CDSs, percentage of fully supported CDSs, and percentage of CDSs for which more than 5% of their sequence is annotated using ab initio prediction.
